# Calcium isotopic ecology of Turkana Basin hominins

**DOI:** 10.1038/s41467-020-17427-7

**Published:** 2020-07-17

**Authors:** Jeremy E. Martin, Théo Tacail, José Braga, Thure E. Cerling, Vincent Balter

**Affiliations:** 1 CNRS, ENSL, LGL-TPE, Univ Lyon, Univ Lyon 1, F-69007 Lyon, France; 2 Bristol Isotope Group, School of Earth Sciences, University of Bristol, Bristol, BS8 1RJ UK; 3 Evolutionary Studies Institute, University of the Witwatersrand, PO Wits, Johannesburg, 2050 South Africa; 4 CNRS UMR 5288, University of Paul Sabatier, 37 Allées Jules Guesde, 31000 Toulouse, France; 5 Department of Geology and Geophysics, University of Utah, Salt Lake City, UT USA

**Keywords:** Palaeoecology, Stable isotope analysis, Biological anthropology, Palaeontology

## Abstract

Diet is a major driver of hominin evolution, but most of the geochemical evidence relies on carbon isotopes (δ^13^C). Here, we report enamel stable calcium isotope (δ^44/42^Ca) values against δ^13^C values for several hominins and co-existing primates in the Turkana Basin area, circa 4 to 2 Ma. *Australopithecus anamensis* clusters with mammal browsers, *Kenyanthropus platyops* is distinct from *A*. *anamensis* in foraging into more open environments and the coexisting *Theropithecus brumpti* encompasses both the grazer and omnivore/carnivore domains. Early *Homo* is remarkable for its wide distribution in δ^44/42^Ca values, possibly reflecting omnivorous and opportunistic preferences. *Paranthropus boisei* is uniquely distributed in the δ^13^C versus δ^44/42^Ca iso-space being distinct from all other hominins from the Turkana Basin area as well as from the co-existing *Theropithecus oswaldi*. Several hypotheses are explored to discuss the unique δ^44/42^Ca values of *Paranthropus boisei* including significant differences observed with δ^44/42^Ca values recently reported for *P*. *robustus* from South Africa, questioning the monophyly of this genus.

## Abstract (web summary)

Non-traditional stable isotopes, such as of calcium, have potential to expand our understanding of ancient diets. Here, Martin et al. use stable calcium isotopes recovered from fossil tooth enamel to compare the dietary ecology of hominins and other primates in the Turkana Basin 2-4 million years ago.

## Introduction

Non-traditional stable isotopes are providing new avenues of research for exploring the ecology, physiology, and dietary preferences of extinct organisms, including our own lineage. Current evidence for early hominin diet relies on comparative osteology, dental tribology, and geochemistry^1–10^. But in the latter case, collagen nitrogen and its isotopes, a well-used trophic level proxy, are rarely preserved in such ancient (i.e., >1 Ma) contexts. While non-traditional isotopes emerge as a new toolkit to paleodietary inference^11–15^, a great deal of the geochemical evidence for early hominin diets relies on carbon isotopes^1–9^ and to a lesser extent on trace element concentrations preserved in mineralized tissues such as fossil bone or teeth^16–19^. The carbon isotopic composition of bioapatite reflects the photosynthetic pathway (i.e., C_3_, C_4_, or CAM) of the ultimate plant source and has been useful for reconstructing past vegetation evolution^20^ and exploring the feeding ecology of African hominins and associated faunas. African hominins cover the whole C_3_–C_4_ spectrum. For example, in East Africa, early australopithecines derived most of their food from C_3_ sources^1,4,6^ whereas more recent forms such as *Paranthropus boisei* almost exclusively relied on C_4_ sources^3,5^. However, significant proportions of C_4_ sources are recorded in some early forms such as *Australopithecus afarensis* or *Kenyanthropus platyops*^1,2,7^. In Central Africa, *Australopithecus bahrelghazali* represents another example of an early hominin that was sourcing its food in a C_4_ environment^8^. Another significant result of carbon isotope studies is the marked difference in δ^13^C values between megadont australopithecines, providing evidence that the South African *Paranthropus robustus* and the East African *Paranthropus boisei* were exploiting different resources^9,17^. Such results were independently corroborated with microwear texture^10,21^. Nonetheless, other proxies are needed to infer hominin diet beyond the ultimate plant source. Here, we investigate resource use in Turkana Basin hominins from Kenya using calcium and carbon isotopes and discuss their relevance for inferring the evolution of dietary habits in the hominin lineage

Seminal studies on calcium isotopes in vertebrates^22^ recognized a decrease in the ^44^Ca/^42^Ca ratio with increasing trophic level. Subsequent studies supported this trophic level effect, notably in the marine realm^23,24^, but also raised awareness on individual physiological variability influencing Ca isotope variability^25,26^ or on the insensitivity of Ca isotope ratios to distinguish between primary and secondary consumers^27^. However, the constant δ^44/42^Ca offset of about −0.6‰ from dietary Ca to bone^22,28^ supports the idea of a propagation-related calcium isotope fractionation from one trophic level to another. This pattern reflects a shared physiological feature of all vertebrates including terrestrial and aquatic mammals, as measured in six different species of mammals including foregut and hindgut digestive physiologies (as reviewed in^14^). Moreover, this trophic level prediction recently found support as measured in a variety of modern and extinct terrestrial faunas^29,30^. Yet, disentangling dietary sources remains challenging because dietary items possess highly variable calcium concentrations and calcium isotopic compositions as exemplified by plants (as compiled in^14,29^), bone^31^ or milk^28,32^. Notably, this last source is ^44^Ca-depleted^28^ and analyzing tissues mineralizing during the pre- to postnatal transition has proved useful to study nursing in modern and fossil hominins^32,33^. Therefore, the isotopic composition of a consumer’s tissue is controlled by the mass-weighted isotopic average of all the dietary sources and in our present case, we do not include data from early forming teeth (e.g., first molars). In other words, whether ^44^Ca-enriched or ^44^Ca-depleted, a narrow range of calcium isotopic composition in a consumer tissue may reflect a specialized diet (e.g., late forming teeth of *Gorilla*, see below and Supplementary Data 1) whereas a scattered distribution should reflect a more complex interplay between resource diversity, isotopic variation in resources, and differential consumption of resources by individuals within a group. As explained above, we favor the hypothesis of a Ca isotope variability primarily related to diet but other aspects such as species-dependent physiologic parameters^25,26^ will have to be further explored in future calcium isotope mammal studies. Therefore, in mammals, calcium isotopes can be used to monitor nursing in early formed teeth^28,32^; later formed teeth can provide information on the intake of calcium (Ca) from adult dietary sources^29^.

Here, we report Ca isotope values for several early hominin species and associated nonhuman primates from the Turkana Basin area. We infer a unique trophic ecology and/or physiology for *Paranthropus boisei* according to its distinct isotopic distribution.

## Results & discussion

### Ca isotopic variability in modern *Gorilla* teeth

**Fig. 1:**
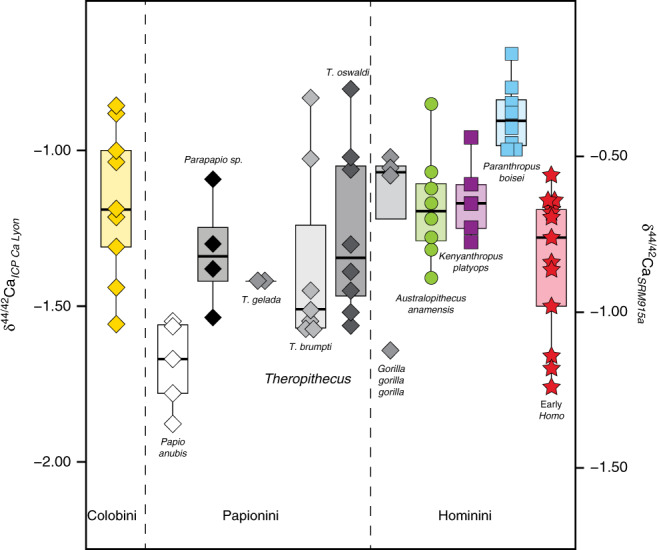
Box and whisker plots showing the distribution of δ^44/42^Ca values (in ‰) between the various non-hominin and hominin primates analyzed in this study as well as some modern representatives (*Papio anubis*, *Theropithecus gelada,* and *Gorilla gorilla gorilla*). Note the ^44^Ca-enriched isotope values of *Paranthropus boisei* in comparison to other groups. The boxes represent the first and third quartiles with the medians as horizontal lines. The lower and upper whiskers represent 1.5 * the interquartile range (numbers of biologically independent samples per group: *n* = 9 for Colobini; *n* = 5 for *Papio anubis*; *n* = 4 for *Parapapio* sp.; *n* = 2 for *Theropithecus gelada*; *n* = 7 for *Theropithecus brumpti*; *n* = 8 for *Theropithecus oswaldi*; *n* = 4 for *Gorilla gorilla gorilla*; *n* = 8 for *Australopithecus anamensis*; *n* = 5 for *Kenyanthropus platyops*; *n* = 8 for *Paranthropus boisei*; *n* = 13 for early *Homo*). Welch’s one-way ANOVA and Kruskal–Wallis tests show significant differences of average means (Welch’s one-way ANOVA on all groups except the two *T*. *gelada* individuals: 10 groups with a total of 71 biologically independent individual samples, *p* value < 10^−4^, *F* = 11.7, df = 9; Kruskal–Wallis on all groups: 11 groups with a total of 73 biologically independent individual samples, *p* value = 0.0005, df = 10). δ^44/42^Ca values are expressed both against ICP Ca Lyon (left) and SRM915a (right). Source data are provided as a Source Data file.

Teeth belonging to four modern western lowland gorilla (*Gorilla gorilla gorilla*) individuals from La Lopé National Park, Gabon, analyzed for Ca isotopes^33^, are presented here as a comparison with other hominin and non-hominin primates (Fig. 1 and Supplementary Data 1). Modern gorillas represent an interesting case study so as to test whether a monotonous herbivorous diet is reflected upon the Ca isotopic composition of bioapatite. Two of the sampled teeth were formed early and include an incisor (G72) with a distinctly low δ^44/42^Ca value as well as a first molar (G39) with a surprisingly high δ^44/42^Ca value, being indistinct from the two other late forming gorilla teeth (second and third molars) available (Supplementary Data 1). The first molar starts mineralization earlier than the incisor (i.e., before birth) and completes mineralization before the third year (within the fourth year for the incisor)^34^. Nevertheless, it should be stressed that tooth enamel from that first molar was sampled for the entire height of the crown, thus mixing enamel formed in utero with enamel formed during or after completion of weaning. Because such teeth mineralize until 3 year of age after birth^35^, weaning and adult food items other than breast milk (in this case plant material) would have contributed to the high δ^44/42^Ca values observed here^36^. It should also be added that dental development is genetically controlled and the huge genetic diversity reported in western lowland gorillas^37^ suggests that a wider spectrum of mineralization patterns exists beyond the published ages reconstructed from histological studies. Therefore, that the first molar (G72) did mineralize later than expected from the dental development models would not be surprising. The two late forming teeth (G40 and G41) display ^44^Ca-enriched compositions and are interpreted as reflecting the post-weaning period of tooth formation during which the individuals were taking their Ca from adult food^33^. A detailed survey of Ca isotope variability in gorilla teeth and other modern primates will certainly bring interesting results to discuss physiological versus dietary influence on Ca isotopic fractionation processes, as currently investigated in humans^26,32,38^.

### Ca isotopic variability in non-hominin fossil primates

**Fig. 2:**
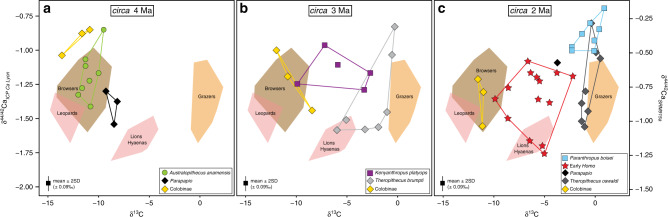
δ^44/42^Ca (in ‰) as a function of δ^13^C (in ‰) measured from tooth enamel of fossil hominin and non-hominin primates from the Turkana Basin area, Kenya for different time bins, i.e., circa 4 Ma, circa 3 Ma and circa 2 Ma (numbers of biologically independent samples per group: *n* = 9 for Colobini; *n* = 4 for *Parapapio* sp.; *n* = 7 for *Theropithecus brumpti*; *n* = 8 for *Theropithecus oswaldi*; *n* = 8 for *Australopithecus anamensis*; *n* = 5 for *Kenyanthropus platyops*; *n* = 8 for *Paranthropus boisei*; *n* = 13 for early *Homo*). The colored areas represent the domains for previously analyzed extant terrestrial mammal enamel from the Turkana Basin area^29^ (*n* = 4 for leopards; *n* = 10 for lions and hyaenas; *n* = 24 for browsers; *n* = 18 for grazers). δ^44/42^Ca values are expressed both against ICP Ca Lyon (left) and SRM915a (right). The average 2SD of each Ca isotopic value is represented at the bottom left of each graph. Source data are provided as a Source Data file.

Our stable calcium isotope values for various hominins and co-existing primates from the Turkana Basin area (*n* = 69) range between −0.69‰ and −1.88‰ (Figs. 1 and 2; Supplementary Data 1). Recently published calcium isotope data for coeval large mammals were shown to preserve their pristine composition, as discussed against elemental concentrations of diagenetic origin of the same samples^29^ (see also Methods). This dataset presents a similar ordering between modern and fossil faunas but also some differences for certain taxa such as suids and saber-tooth cats, interpreted to have had changing dietary ecologies through time^29^.

Papionines have often been considered as analogues to understand dietary preferences of early hominins^1^. The ^44^Ca-depleted isotope values of modern *Papio anubis* (Fig. 1) are to be considered under the light of the broad feeding preferences in cercopithecines^39,40^. In comparison, the more positive Ca isotope values of *Parapapio* sp. invite to consider that extinct taxa may not have been similar in their feeding preferences, although one must take into account that modern *Papio anubis* has a wide distribution in Africa today and our data cannot reflect such spatial variations. Modern geladas do intensively feed on high altitude C_3_ grasses^41^ and our δ^44/42^Ca values (Supplementary Data 1) for two modern grass-eating *Theropithecus gelada* (−1.40‰) are consistent with a grass-eating ecology. Grazers are ^44^Ca-depleted relative to leaf-eaters by about 0.3‰ and other modern grazers such as zebras, buffaloes, warthogs, and hippos also display comparably low calcium isotope values that overlap with carnivore values^29^ (Fig. 2). Morphological evidence shows grass-eating adaptations in extinct *Theropithecus*^42^; carbon isotopes confirm an early dietary adaptation to C_4_ grasses as an important component of their diet^43^. Here, δ^44/42^Ca values for *T*. *brumpti* (−1.36 ± 0.31‰, 1SD, *n* = 7) and *T*. *oswaldi* (−1.26 ± 0.27‰, 1SD, *n* = 8) are dispersed indicating the availability of a range of isotopically different calcium sources consumed in different proportions by different individuals in each species. Some of those values are not departing much from those of the modern *T*. *gelada*, and a grass-eating ecology is a probable dietary hypothesis for some individuals of both fossil species. Nevertheless, this does not exclude other food sources, for example as illustrated here with ^44^Ca-depleted values for several individuals of *T*. *brumpti* falling within the same range of values as those of large carnivores (Fig. 2b). *T*. *brumpti* was a large-sized theropithecine compared with *T*. *oswaldi* or to the extant *T*. *gelada* and contrary to those, *T*. *brumpti* was clearly foraging into mixed environments according to δ^13^C values. Given the wide range of δ^44/42^Ca values observed in *T*. *brumpti*, an omnivorous diet, sometime including bone, is plausible.

### Ca isotopic variability in fossil hominins

*Australopithecus anamensis* and *Kenyanthropus platyops* could not be distinguished on the basis of calcium isotope values. *K*. *platyops* carbon isotope values clearly showed that it foraged in mixed C_3_-C_4_ environments whereas values for *A*. *anamensis* indicate a nearly pure-C_3_ diet^1^ similar to *Ardipithecus ramidus*^4^. Although early studies based on cranial morphology suggested tough/abrasive food items in the diet of *A*. *anamensis*, more recent studies based on microwear and carbon isotopes proposed a diet of soft plant items from the C_3_ environment^1,44^. Here, the distribution of calcium isotope values in *A*. *anamensis* (−1.18 ± 0.17‰, 1SD, *n* = 8) is not distinctly ^44^Ca-depleted and overlaps much of the modern and fossil East African browser domains (Fig. 2) and indicates that it was foraging on a variety of C_3_ resources, perhaps fruits or plants, as previously suggested from carbon isotopes^1^. Some individuals of *A*. *anamensis* may have spent some time in open habitats as indicated by positive δ^18^O values, clearly distinct from the ^18^O-depleted values of *Parapapio* sp. and colobines (Supplementary Fig. 1, Supplementary Data 1), suggesting a decoupling between food and habitat use.

The δ^44/42^Ca values in the early *Homo* group are dispersed (−1.36 ± 0.23‰, 1SD, *n* = 13) and may either indicate wide dietary flexibility, incorporating different sources with varied calcium isotopic values and concentrations, possibly including omnivorous or carnivorous preferences for the most ^44^Ca-depleted isotope values. In addition, such values may reflect a lack of taxonomic resolution within a diversified early *Homo* group in the Turkana Basin area containing *Homo habilis*, *Homo rudolfensis*, and *Homo erectus*^45^, and could be due to heterogeneous dietary habits within the genus *Homo*. Also notable is that early *Homo* (−1.36 ± 0.23‰, 1SD, *n* = 13) and *Theropithecus oswaldi* (−1.26 ± 0.27‰, *n* = 8) have similar calcium isotope values but not carbon isotope values.

### The peculiar case of *Paranthropus boisei*

*Paranthropus boisei* displays the most ^44^Ca-enriched values of the dataset (−0.89 ± 0.11‰, 1SD, *n* = 8) being statistically different from early *Homo* (Welch’s *t*-test, p*** < 10^−4^), *Theropithecus oswaldi* (Welch’s *t*-test, p** = 0.006) and from Turkana mammal grazers (Welch’s *t*-test, p*** < 10^−4^). That *P*. *boisei* values do not overlap in a δ^13^C versus δ^44/42^Ca space with any other hominins and non-hominin grazers (Figs. 1 and 2; Supplementary Data 2) adds to the uniqueness of this taxon, as previously highlighted in the literature with morphological, microwear, or carbon isotopic studies.

*P. boisei* possesses a unique tooth morphology with thick enamel and flatten occlusal surfaces when worn, and dental microwear studies do suggest a diet of soft plant items^21^. If leaves appear to be a good dietary candidate, C_4_ leaves other than blades from grasses or sedges are not obviously identified in modern ecosystems. *Theropithecus gelada* cannot be considered a modern primate analogue for *P*. *boisei*, given their differences in δ^44/42^Ca values. A modern primate analogue for *P*. *boisei* that would feed on C_4_ vegetation other than grass or sedge does not exist. Gorillas could be viewed as a remote plant-eating analogue. The two late-forming modern western lowland gorilla teeth from La Lopé in Gabon display calcium isotope values around −1.05‰ (Supplementary Data 1)^33^, comparable with the values of *P*. *boisei*. Although those gorillas feed on fruits, leaves, and ants, these resources arise from C_3_, not C_4_ plants^11^. While dicot leaves and fruits are ^44^Ca-enriched, the only dicot leaves that follow the C_4_ photosynthetic pathway are represented by forbs, which would be found in relatively wet environments^46^ and could be a dietary candidate for *P*. *boisei* according to oxygen isotope composition of *P*. *boisei* teeth suggesting water dependency^5^. Conducting a calcium isotope survey of such plants but also of C_4_ sedges, especially in including plant parts that are known to fractionate differently^47^, would certainly add relevant data to better tackle this problem. On the other hand, we cannot exclude that both *G. gorilla* and *P. boisei* had physiological similarities that would lead to ^44^Ca/^42^Ca values that are enriched relative to other primates.

**Fig. 3:**
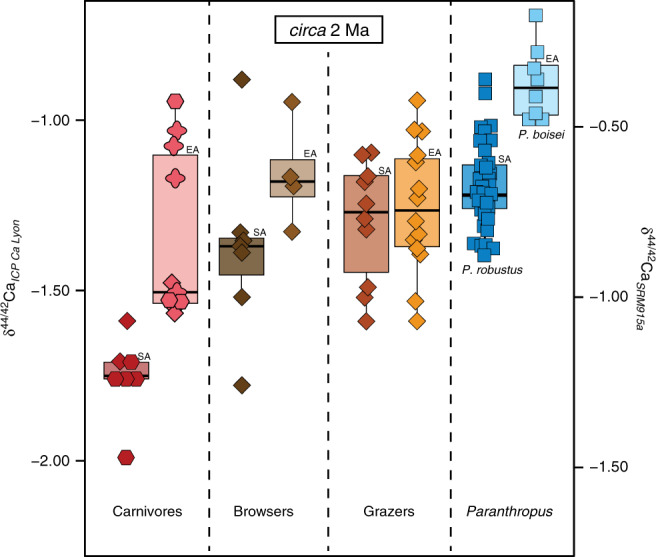
Box and whisker plots showing the distribution of δ^44/42^Ca values (in ‰) between Turkana Basin *Paranthropus boisei*, South African *Paranthropus robustus*^33^ and associated mammal faunas. Data for grazers, browsers, and carnivores are from Martin et al.^29^ and Tacail et al.^33^. EA East Africa, SA South Africa. Values selected for representatives of the associated-fauna include only those values for organisms circa 2 Ma; for this reason, the browser category may appear underrepresented and the apparent difference may reflect specific dietary differences rather than differences in the substrate isotope baseline. Note that carnivore values in EA are dispersed as explained by a dataset that includes sabertooth felids. The boxes represent the first and third quartiles with the medians as horizontal lines. The lower and upper whiskers represent 1.5 * the interquartile range (numbers of biologically independent samples per group: *n* = 7 for South African carnivores; *n* = 10 for East African carnivores; *n* = 7 for South African browsers; *n* = 4 for East African browsers; *n* = 10 for South African grazers; *n* = 14 for East African grazers; *n* = 38 for *Paranthropus robustus*; *n* = 8 for *Paranthropus boisei*). δ^44/42^Ca values are expressed both against ICP Ca Lyon (left) and SRM915a (right). Source data are provided as a Source Data file.

Whatever the exact dietary component or physiological specificity of *P*. *boisei* was, this species remains remarkable for exhibiting ^44^Ca-enriched tooth enamel within a narrow range of isotope values, which most likely reflect a specialized diet on items with low isotopic variability^48^ possibly associated to a physiology different than other primates. The range of calcium isotope values of *P*. *boisei* not only differs from other hominins of the Turkana Basin, it also differs from that of its South African congener *P*. *robustus*, which shows lower calcium isotope values^33^ (Fig. 3). Interestingly, *P*. *robustus* and *A*. *africanus* do not differ in δ^13^C values^9^, nor do they differ in δ^44/42^Ca values^33^ (Fig. 3). *P*. *robustus* was interpreted to have had a flexible diet together with the contemporaneous early *Homo* from South Africa^9,49^, a hypothesis that seems corroborated by the overlap of calcium isotope values of *P*. *robustus* with early *Homo* and *A*. *africanus*^33^. From our study as well as previous ones based on δ^13^C values^1^ or tooth microwear^21^, the emerging picture could be that of *P*. *boisei* in East Africa with a specialized diet versus *P*. *robustus* in South Africa with a flexible diet^9,49^. These results confirm discrepancies between diets but apparently shared masticatory function and weaken the case for *Paranthropus* monophyly based solely on dentognathic features^50^.

In summary, calcium isotope ratios in a survey of hominin lineages in East Africa show that *P. boisei* has a unique ^44^Ca/^42^Ca ratio compared with other hominins including the South African *P. robustus*, all of which have similar ratios to each other but not to *P. boisei*. This calcium isotope difference could be due to differences in diet, or in physiology, and remains a unique character of *P. boisei* compared with other hominins.

## Methods

### Fossil samples

Fossil samples were available from the collections of the National Museums of Kenya and the Turkana Basin Institute. Those fossil samples are part of a paleoecology project reported in Cerling et al.^51^. Powdered enamel was collected using a low-speed dental drill along the broken surfaces of tooth enamel. Each sample represents an average of the crown height in order to provide enough material for the published carbon isotope analyses initially envisioned. 100–200 µg of powder per sample was used for Ca isotope purification.

### Modern samples

Two geladas (*Theropithecus gelada*) individuals were made available from the historical collections of the Musée des Confluences in Lyon, France (MHNL). The precise Ethiopian origin of the specimens is not provided on the labels. Specimen MHNL 50001812 is a male individual and was donated to the Museum by Claudius Côte on the 13th of august 1929. Specimen MHNL 50001729 is a male individual from Abyssinia and was bought from Mr. Siepi, then a naturalist in Marseille on the 3rd of march 1925.

### Sample chemistry

The chemical processing of the samples follows the method described in details in Martin et al.^29^ and Tacail et al^33,52^. Briefly, enamel samples were dissolved in 300 μl of suprapure 1 M HCl acid and were subsequently processed through AG50X-W12 cation exchange resin in 1 M HCl medium to dispose of sample matrix. Ca and Sr fractions were collected in 6 M HCl medium. Ca fractions were then separated from Sr by loading samples onto columns filled with Sr-specific resin (Eichrom Sr-Spec) in suprapure 2 M HNO_3_ medium. Blanks for the whole procedure did not exceed 100 ng Ca.

### Isotopic measurements

Calcium isotope abundance ratios (^44^Ca/^42^Ca and ^43^Ca/^42^Ca) were measured using a multi-collector ICP-MS (MC-ICP-MS, Neptune Plus, Thermo). After purification, Ca samples were dissolved in ultrapure 0.05 M HNO_3_ and Ca concentration was set at 2 ppm for all samples and standards. All Ca isotope compositions are expressed using the ‘delta’ notation defined as follows for the ^44^Ca/^42^Ca ratio:

1 $$\delta ^{44/42}{\mathrm{Ca}} \,=\, \left[ {\frac{{\left( {{\,\!}^{44}{\mathrm{Ca}}/{\,\!}^{42}{\mathrm{Ca}}} \right)_{\mathrm{{sample}}}}}{{\left( {{\,\!}^{44}{\mathrm{Ca}}/{\,\!}^{42}{\mathrm{Ca}}} \right)_{\mathrm{ICP}\,{\mathrm{Ca}}\,{\mathrm{Lyon}}}}} - 1} \right] \times 1000,$$

where (^44^Ca/^42^Ca)_sample_ and (^44^Ca/^42^Ca)_ICP Ca Lyon_ are the Ca isotope abundance ratios measured in sample and ICP Ca Lyon reference standard, respectively. The ICP Ca Lyon standard, used as a bracketing standard, is a Specpure Ca plasma standard solution (Alfa Aesar)^12,24,30,32,33,52–55^. All newly reported samples were measured as part of the same batches as the samples reported in Martin et al.^29^, i.e., in June and August 2016 and in June and July 2017. A single concentrated solution of NIST SRM 1486 was repeatedly purified and measured in the same batches as the samples to control for accuracy. In order to ease comparison of our dataset with other Ca isotope studies from the literature, all values obtained in this study are also expressed relative to SRM915a standard (Supplementary Data 1) using the constant difference of −0.518 ± 0.025‰ (2SD) as calibrated by analyzing four international standards previously reported against SRM915a and repeatedly measured against ICP Ca Lyon (see Supplementary Information in Martin et al.^29^). All statistical tests on the Ca isotope data were performed with the R “stats” package^56^.

### Quality

Samples were randomly measured during the ICP-MS sessions that served to build the dataset published on the modern and fossil Turkana fauna in Martin et al.^29^. All samples fall on a calcium mass fractionation line with a slope of 0.519 ± 0.018‰ (2SE) (Supplementary Fig. 2) in good agreement with the 0.5067 slope predicted by the linear approximation of exponential mass-dependent fractionation. Independent measurements of NIST SRM 1486 standards yielded a mean value of: −1.047 ± 0.13‰ 2 SD (*n* = 101). These values are the same as those measured in the same laboratory at LGLTPE^12,24,30,32–34,52–55^ and agree well with previously published values from other laboratories^31,57–59^.

### Diagenesis

It is well known that diagenetic processes can alter the original isotopic composition of mineralized tissues. That Ca is a major constituent of bioapatite (at a concentration of about 35–40% in weight) represents a solid reason to investigate its isotopic composition in fossil bone and teeth as old as the Cretaceous^30,31,53^. Here, before chemical purification of Ca isotopes, all samples underwent the standard protocol that is applied for carbon isotopic analyses by removing potential secondary carbonates^51^. Measuring trace element concentrations requires more than half a milligram—when such elements are present—and therefore necessitates large quantities of sample powder at the cost of precious samples, such as the hominin teeth analyzed in the present study. In this study, our average sample weight uptake was about 100 µg, which is suitable for Ca isotope analysis but obviously not for trace element concentrations. Correlations between trace elements of diagenetic origin and Ca isotopic compositions were assessed in a previous study of fossil mammals from the same Turkana Basin localities^29^ as those of the hominins analyzed in the present study. We concluded that the impact of diagenesis on our Ca isotopic measurements was minimal^29^ and by extension, we consider such conclusions applicable to the present hominin dataset.

### Comparing East African versus South African datasets

Because a substrate effect cannot be excluded on the fractionation of calcium isotopes, we used associated faunal calcium isotope compositions^29,33^ to serve as a baseline. This issue has not been explored in detail yet^29^ but it is worth repeating here that Ca isotopic composition in rocks is rather homogenous^60^ and a substrate effect may not be pre-eminent in vertebrate tissues. The Turkana fossil assemblage circa 2 Ma includes six felids and four hyaenids calcium isotope values more variable than the range of values of the seven carnivores from South Africa that also includes felids and hyaenids. This dispersion in Ca isotope values is explained by the presence in the Turkana dataset of saber tooth felids, whose diet was specialized as previously discussed^29^. The fossil browsers from Turkana (–1.16‰, *n* = 4) versus browsers from South Africa (–1.38‰, *n* = 7) show different values. Browsers from South Africa consist of one genus only, *Tragelaphus*, which is known in modern South Africa to include about one-third of C_4_ component in its diet^61^ and might explain some of its values to be ^44^Ca-depleted. The low number of browsers from East Africa that includes two giraffids and two bovids does not permit to satisfyingly compare both browser datasets. Finally, the fossil grazers consist of a larger dataset; taxa from Turkana (–1.25‰, *n* = 14) versus grazers from South Africa (–1.30‰, *n* = 10) gave consistent values indicating that beyond some variability that may arise from seasonal variation into food items (although this is restrained by the sampling protocol-see above), nursing processes or other unconstrained environmental processes, it is reasonable to compare both South African and East African palaeoenvironments.

### Reporting summary

Further information on research design is available in the Nature Research Reporting Summary linked to this article.

## Supplementary information

Supplementary Information

Reporting Summary

Description of Additional Supplementary Files

Supplementary Data 1

Supplementary Data 2

## Data Availability

The authors declare that all data supporting the findings of this study are included in this published article and its supplementary information files. Source data are provided with this paper. Ca, O and C isotope data of modern and fossil non-primates used in Fig. 2 and Supplementary Figs. 2 and 3 are available from the public repository HAL at https://hal-udl.archives-ouvertes.fr/hal-02568580/. Ca isotope data of fossil taxa from South Africa used in Fig. 3 are available from the open access dataset at https://advances.sciencemag.org/content/5/8/eaax3250/tab-figures-data. Source data are provided with this paper.

## Source data

Source Data
