# Supplementary material for: Calcium isotopic ecology of Turkana Basin hominins
Source: Nat Commun. 2020 Jul 17;11:3587. doi: 10.1038/s41467-020-17427-7 (PMC7367883; doi:10.1038/s41467-020-17427-7)
Supplement: Supplementary file 1 — Supplementary Information [file 41467_2020_17427_MOESM1_ESM.pdf]

Supplementary Information

Calcium isotopic ecology of Turkana Basin hominins

Martin et al.

This file comprises:

Supplementary Figure 1: $\delta^{44/42}$Ca as a function of δ^{18}O measured from tooth enamel of fossil hominin and non-hominin primates from the Turkana Basin area, Kenya for different time bins, i.e. circa 4 Ma (A), circa 3 Ma (B) and circa 2 Ma (C).

Supplementary Figure 2: Three-isotope-plot for all data measured in this work and in Martin et al. [1] (n=164) showing $\delta^{43/42}$Ca (‰) as a function of $\delta^{44/42}$Ca (‰) relative to *ICP Ca Lyon* bracketing standard and SRM915a.

Supplementary Figure 3: $\delta^{44/42}$Ca as a function of δ^{13}C compared between the modern mammalian assemblage of Tsavo National Park and the fossil mammalian assemblage from Turkana Basin, Kenya.

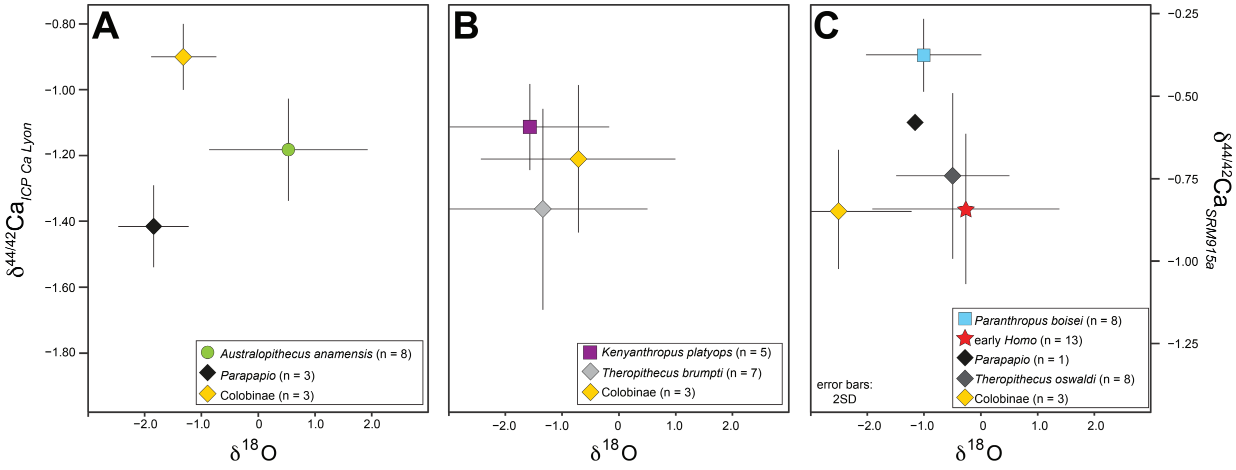

Supplementary Figure 1. $\delta^{44/42}$Ca as a function of δ^{18}O measured from tooth enamel of fossil hominin and non-hominin primates from the Turkana Basin area, Kenya for different time bins. Circa 4 Ma (A), circa 3 Ma (B) and circa 2 Ma (C). $\delta^{44/42}$Ca values are expressed both against ICP Ca Lyon (left) and SRM915a (right). Error bars correspond to 2SD computed from the average value ($\delta^{44/42}$Ca and δ^{18}O) of each taxon group. Source data are provided as a Source Data file (the numbers of biologically independent samples per group are given in the legends of each graph).

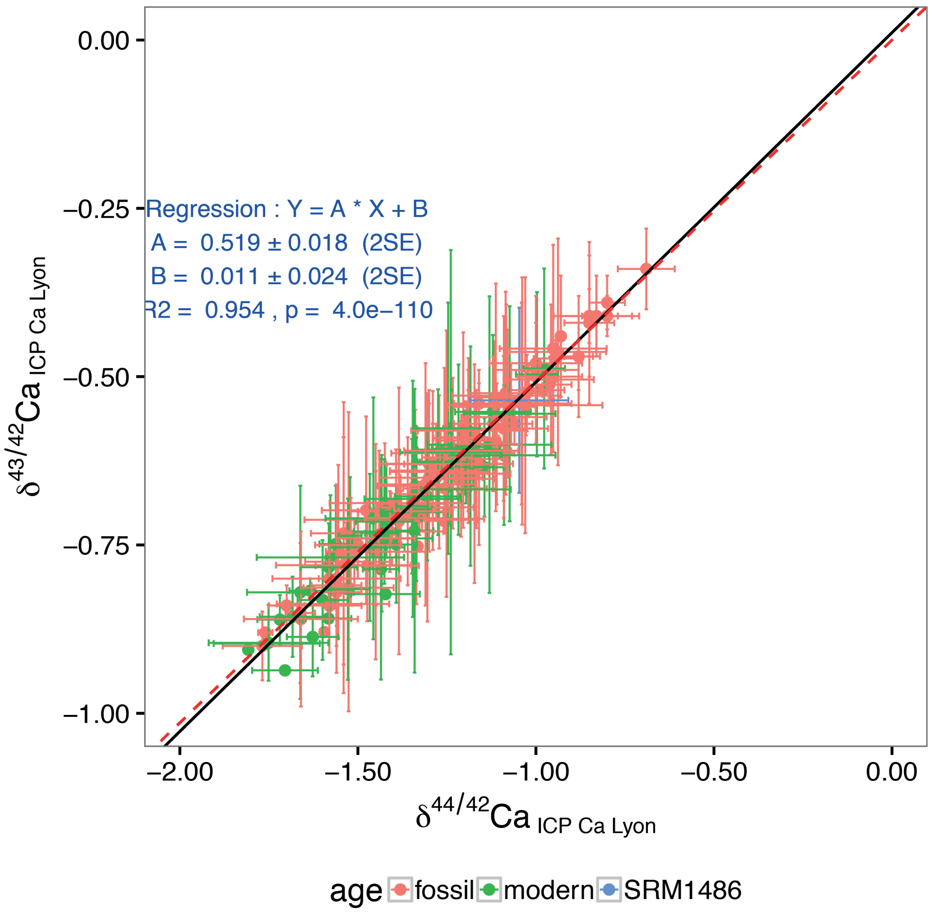

Supplementary Figure 2. Three-isotope-plot for all data measured in this work and in Martin et al. [1] **(n=164 biologically independent samples) showing $\delta^{43/42}$Ca (‰) as a function of $\delta^{44/42}$Ca (‰) relative to *ICP Ca Lyon* bracketing standard and SRM915a.** Error bars on every single Ca isotope values correspond to 2SD computed from analytical replicates. The linear regression was performed with the R "stats" package ("lm" function) and corresponds to a linear least squares model (one-sided), for which all results are given in blue on the graph. The black solid line corresponds to the linear regression while the red dashed line represents the theoretical line with the 0.507 slope predicted by the exponential law linear approximation of mass-dependent fractionation. Source data are provided as a Source Data file.

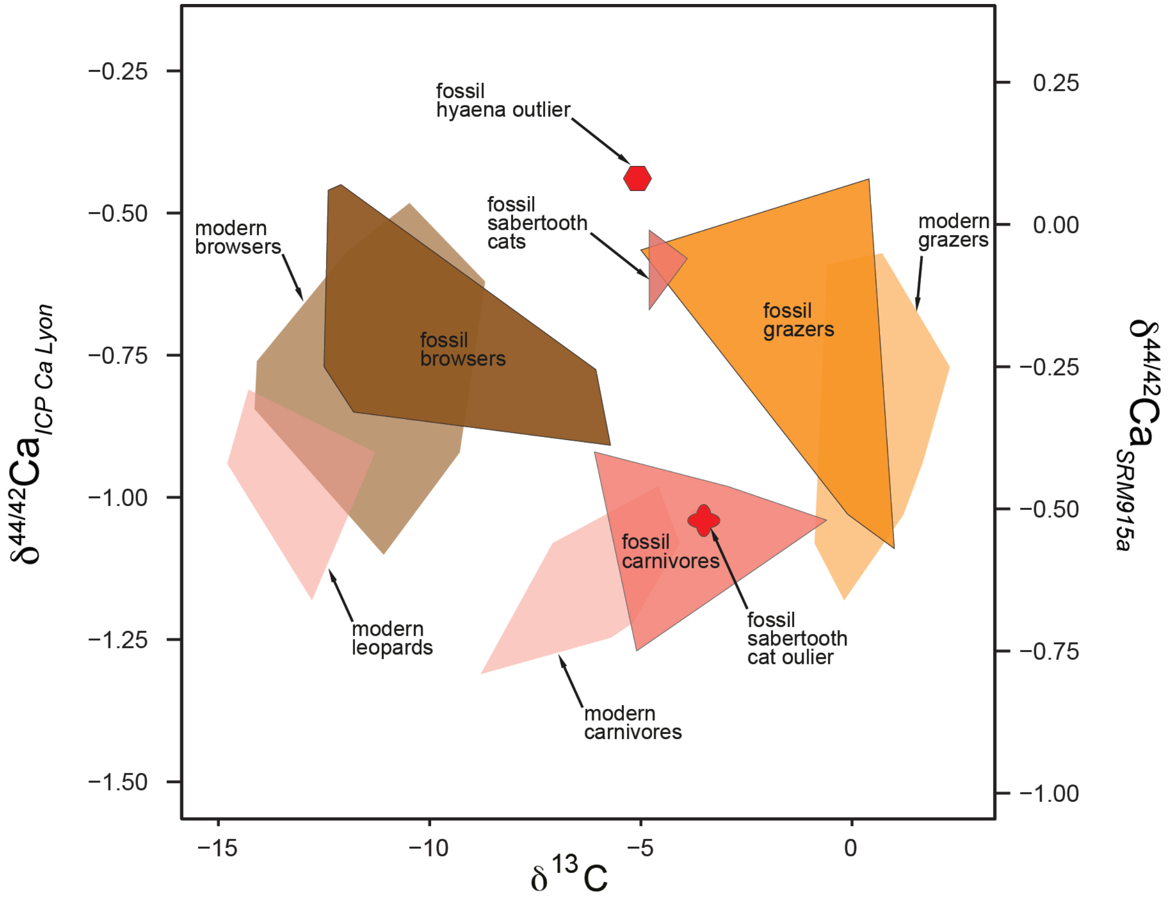

Supplementary Figure 3. $\delta^{44/42}$Ca as a function of δ^{13}C compared between the modern mammalian assemblage of Tsavo National Park and the fossil mammalian assemblage from Turkana Basin, Kenya. This display shows that the main iso-ecological domains for mammal grazers, browsers and most carnivores to be similar in both modern and fossil contexts. Although data are preliminary, saber-tooth cats, an extinct lineage differing in their ecology from modern felids, represent the exception. Data after Martin et al. [1]. $\delta^{44/42}$Ca values are expressed both against ICP Ca Lyon (left) and SRM915a (right). (numbers of biologically independent samples per group: n=4 for modern leopards; n=10 for modern carnivores; n=24 for modern browsers; n=18 for modern grazers; n=9 for fossil carnivores; n=3 for fossil sabertooth cats; n=8 for fossil browsers; n=24 for fossil grazers). Source data are provided as a Source Data file.

Supplementary References

1- Martin, J. E., Tacail, T., Cerling, T. E., & Balter, V. Calcium isotopes in enamel of modern and Plio-Pleistocene East African mammals. *Earth Planet. Sci. Lett.* **503**, 227–235 (2018).
