# Supplementary material for: Calcium isotopic ecology of Turkana Basin hominins
Source: Nat Commun. 2020 Jul 17;11:3587. doi: 10.1038/s41467-020-17427-7 (PMC7367883; doi:10.1038/s41467-020-17427-7)
Supplement: Supplementary file 2 — Reporting Summary [file 41467_2020_17427_MOESM2_ESM.pdf]

nature research

Corresponding author(s): Jeremy E. Martin

Last updated by author(s): Jun 23, 2020

Reporting Summary

Nature Research wishes to improve the reproducibility of the work that we publish. This form provides structure for consistency and transparency in reporting. For further information on Nature Research policies, see Authors & Referees and the Editorial Policy Checklist .

Statistics

For all statistical analyses, confirm that the following items are present in the figure legend, table legend, main text, or Methods section.

n/a	Confirmed	
☐	☒	The exact sample size (n) for each experimental group/condition, given as a discrete number and unit of measurement
☐	☒	A statement on whether measurements were taken from distinct samples or whether the same sample was measured repeatedly
☐	☒	The statistical test(s) used AND whether they are one- or two-sided<br>*Only common tests should be described solely by name; describe more complex techniques in the Methods section.*
☒	☐	A description of all covariates tested
☒	☐	A description of any assumptions or corrections, such as tests of normality and adjustment for multiple comparisons
☐	☒	A full description of the statistical parameters including central tendency (e.g. means) or other basic estimates (e.g. regression coefficient) AND variation (e.g. standard deviation) or associated estimates of uncertainty (e.g. confidence intervals)
☐	☒	For null hypothesis testing, the test statistic (e.g. F, t, r) with confidence intervals, effect sizes, degrees of freedom and P value noted<br>*Give P values as exact values whenever suitable.*
☒	☐	For Bayesian analysis, information on the choice of priors and Markov chain Monte Carlo settings
☒	☐	For hierarchical and complex designs, identification of the appropriate level for tests and full reporting of outcomes
☒	☐	Estimates of effect sizes (e.g. Cohen's d, Pearson's r), indicating how they were calculated

Our web collection on statistics for biologists contains articles on many of the points above.

Software and code

Policy information about availability of computer code

Data collection	Neptune plus software package from Thermo Scientific
Data analysis	Microsoft Excel version 16.38, R Core Team (2018). R: A language and environment for statistical computing. R Foundation for Statistical Computing, Vienna, Austria. URL https://www.R-project.org/.

For manuscripts utilizing custom algorithms or software that are central to the research but not yet described in published literature, software must be made available to editors/reviewers. We strongly encourage code deposition in a community repository (e.g. GitHub). See the Nature Research guidelines for submitting code & software for further information.

Data

Policy information about availability of data

All manuscripts must include a data availability statement. This statement should provide the following information, where applicable:

- Accession codes, unique identifiers, or web links for publicly available datasets
- A list of figures that have associated raw data
- A description of any restrictions on data availability

The authors declare that all data supporting the findings of this study are included in this published article and its Supplementary Figures. Source data (Table 1) are provided with this paper. Ca, O and C isotope data of modern and fossil non-primates used in Figures 2 and Supplementary Figures 2 and 3 are available from the public repository HAL at https://hal-udl.archives-ouvertes.fr/hal-02568580/the Earth and Planetary Science Letters website (https://www.sciencedirect.com/science/article/abs/pii/S0012821X1830565X). Ca isotope data of fossil taxa from South Africa used in Figure 3 are available from the open access dataset at https://advances.sciencemag.org/content/5/8/eaax3250/tab-figures-data.

Field-specific reporting

Please select the one below that is the best fit for your research. If you are not sure, read the appropriate sections before making your selection.

- [ ] Life sciences
- [ ] Behavioural & social sciences
- [x] Ecological, evolutionary & environmental sciences

For a reference copy of the document with all sections, see nature.com/documents/nr-reporting-summary-flat.pdf

Ecological, evolutionary & environmental sciences study design

All studies must disclose on these points even when the disclosure is negative.

Study description	Calcium isotope purification in the clean lab followed by isotopic measurement on a MC-ICP-MS (Thermo Neptune Plus)
Research sample	Access to fossil tooth enamel of various hominins and non-hominin primates is controlled by the curator. Fossils being rare and precious, only a limited number can be accessed for destructive sampling, even considering a minimally destructive approach. Hence, specimens showing broken tooth surfaces were preferentially selected. Current knowledge about a sample population size for a given hominin species cannot be established but our sampling certainly represents an underestimation (n=8 for Australopithecus anamensis; n=5 for Kenyanthropus platyops; n=14 for early Homo; n=8 for Paranthropus boisei).
Sampling strategy	No statistical method was used, as all samples were considered of interest from the leftover powders used in Cerling et al. (2013) for which carbon isotopes were already available. Therefore, no additional sampling on Museum specimens was necessary as part of this project.<br>Cerling, T. E., Manthi, F. K., Mbua E. N., Leakey L. N., Leakey M. G., Leakey R. E., Brown F. H., Grine F. E., Hart J. A., Kaleme P. & Roche H. Stable isotope-based diet reconstructions of Turkana Basin hominins. Proc. Natl. Acad. Sci. USA 110, 10501–10506 (2013).
Data collection	Calcium isotope abundance ratios (44Ca/42Ca and 43Ca/42Ca) were measured using a multi-collector ICP-MS (MC-ICP-MS, Neptune Plus, Thermo) by JEM, TT, TEC and VB.
Timing and spatial scale	Calcium isotope abundance ratios (44Ca/42Ca and 43Ca/42Ca) were measured as part of the same batches as the samples previously reported in Martin et al. (2018), i.e. in June and August 2016 and in June and July 2017.<br>Martin, J. E., Tacail, T., Cerling, T. E., & Balter, V. Calcium isotopes in enamel of modern and Plio-Pleistocene East African mammals. Earth Planet. Sci. Lett. 503, 227–235 (2018).
Data exclusions	no data were excluded from the analysis
Reproducibility	The reproducibility for the measurement of Ca isotope compositions is assessed through the repeated analysis of bone secondary standard (NIST SRM1486). Standards were repeatedly introduced during chemical purification and their measured values are reported for each analytical session (Independent measurements of NIST SRM 1486 standards yielded a mean δ44/42Ca value of: –1.047 ± 0.13 ‰ 2SD, n = 101).
Randomization	All sampled specimens were assigned a lab number, so no taxonomic identification could be followed during chemical treatment and isotopic analyses. Results were then confronted to the taxonomic id when the analytical procedure was finished.
Blinding	Blinding was not relevant to this study because no treatments were compared

Did the study involve field work? [ ] Yes [x] No

Reporting for specific materials, systems and methods

We require information from authors about some types of materials, experimental systems and methods used in many studies. Here, indicate whether each material, system or method listed is relevant to your study. If you are not sure if a list item applies to your research, read the appropriate section before selecting a response.

Materials & experimental systems

n/a	Involved in the study	
[x]	[ ]	Antibodies
[x]	[ ]	Eukaryotic cell lines
[ ]	[x]	Palaeontology
[x]	[ ]	Animals and other organisms
[x]	[ ]	Human research participants
[x]	[ ]	Clinical data

Methods

n/a	Involved in the study	
[x]	[ ]	ChIP-seq
[x]	[ ]	Flow cytometry
[x]	[ ]	MRI-based neuroimaging

Palaeontology

Specimen provenance	Museum specimens were sampled from the National Museums of Kenya and Musée des Confluences de Lyon, France.<br>National Museums of Kenya: Kenya Research Permit number: NACOSTI/P/18/95520/23873, 18 July 2018 to TEC for the project "Paleoecology and Ecology in Kenya using Stable Isotopes". NACOSTI: National Commission for Science, Technology, and Innovation.<br>Musée des Confluences: Access to samples was authorized to JEM on the 25th of April 2019 by the curator of Musée des Confluences (D. Berthet); the file is archived by Musée des Confluences under "Dossier Scientifique: J. Martin".<br>Curation numbers are available in Table 1.
Specimen deposition	National Museums of Kenya, Kenya and Musée des Confluences de Lyon, France
Dating methods	The present contribution does not employ dating methods.

☐ Tick this box to confirm that the raw and calibrated dates are available in the paper or in Supplementary Information.
