# Supplementary material for: Calcium isotopic ecology of Turkana Basin hominins
Source: Nat Commun. 2020 Jul 17;11:3587. doi: 10.1038/s41467-020-17427-7 (PMC7367883; doi:10.1038/s41467-020-17427-7)
Supplement: Supplementary file 3 — Description of Additional Supplementary Files [file 41467_2020_17427_MOESM3_ESM.pdf]

Description of Additional Supplementary Files

File Name: Supplementary Data 1

Description: Compilation of tooth enamel calcium isotopic data analysed in this work together with new as well as previously published carbon and oxygen isotope values for the same specimens. Abbreviations in "Tooth type" column: lt, left; rt, right; uppercase, upper tooth row; lowercase, lower tooth row.

File Name: Supplementary Data 2

Description: Summary of Ca isotope compositions per group in hominin and non-hominin primates from this present study, as well as non-primate modern and fossil fauna from Marin et al. [29]. The average $d^{44/42}$Ca values are shown together with the results of the parametric Welch's t-test (unequal variances, two-sided, unpaired Student t-test) for the comparison of the mean composition of *P. boisei* with the mean of each other group.
